# SARS-CoV-2 tropism: what urologists need to know

**DOI:** 10.1186/s12301-021-00126-0

**Published:** 2021-02-01

**Authors:** Elsayed Desouky

**Affiliations:** 1 Urology Department, Wexham Park NHS Hospital, 15 Yew Tree Road, Slough, Berkshire, SL1 2AA UK; 2 Urology Department, Alexandria Main University Hospital, Alexandria, Egypt

**Keywords:** COVID-19, Pandemic, SARS-CoV2, Viral infection, Urology

## Abstract

**Background:**

Apart from viral sexually transmitted diseases, viral infections in urology are not common and likely to be underreported. Initially, COVID-19 was thought to be only affecting our practice indirectly through reducing elective work that almost came to a stop. However, recent upcoming reports show that urologists can get involved far beyond that.

**Main body:**

Genitourinary tract can be directly affected based on the SARS-CoV-2 virus organotropism. The aim of this article is to present a comprehensive review of the data available and to highlight any possible similarity with the few known viral infections involving genitourinary organs with regard to its pathophysiologic impact.

**Conclusion:**

Urologists need to extrapolate their experience with viral infections in the urinary tract so as to be able to manage such possible COVID infections and its short- and long-term consequences.

## Background

COVID-19 pandemic continues to overwhelm the globe. Severe acute respiratory syndrome coronavirus 2 (SARS-CoV-2) targets primarily the respiratory tract. Nevertheless, other organs have been found to be involved including the kidneys, liver, heart, and brain. Hence, it can be stated that SARS-CoV-2 has a broad organotropism [1].

Apart from viral STDs, viral infections in urology are not common in our daily practice and symptoms related to viral infections of the urinary tract can be frequently missed or underreported. Therefore, we need to highlight the possibility of dealing with SARS-CoV-2 in our practice.

## Main body

### Kidney

Viral infections are responsible for significant morbidity and mortality in several renal diseases. This can be through one of two pathways of pathogenicity;

1. Viral replication within the renal tissue, e.g., Epstein-Barr virus and cytomegalovirus, is one particular mechanism of pathogenicity, particularly in immunocompromised patients [2].
2. On the other hand, hepatitis C or human immunodeficiency virus (HIV) can cause an indirect injury to the renal tissue through immune complex deposition [2].

Amid the current COVID-19 pandemic, kidneys are found to be among the most common targets of SARS-CoV-2. It was found that RNA for angiotensin-converting enzyme 2 (ACE2) receptor is abundant in the renal tissue. This facilitates SARS-CoV-2-associated kidney injury. Involving all compartments of the kidney, special affinity of SARS-CoV-2 for glomerular cells was confirmed [1].

Based on that, renal tropism is a likely clarification of the clinical findings of kidney injury in patients with COVID-19 even in patients with SARS-CoV-2 disease who are not significantly sick [1].

In a case series of five kidney transplant patients with COVID-19, it was found that severe COVID-19 did not develop although they were immunocompromised. Mild COVID-19 in renal transplant patients can be managed with symptomatic support therapy together with cautious adjustment of their immunosuppressive therapy [3].

### Bladder

Viruses are an uncommon cause of cystitis in an immunocompetent individual. Hemorrhagic cystitis in an immunocompromised patient can be caused by adenovirus, and cytomegalovirus or BK virus. In this case, the antiviral drug of choice is Cidofovir [4].

Regarding SARS-CoV-2, it was found that ACE2 receptor expression was found in about 2.4% of urothelial cells with possible subsequent viral cystitis [5].

Increased urinary frequency has been identified in a small series of some COVID-19 patients with no evidence of urinary tract infection [5].

It is as yet not confirmed whether the ACE 2 receptor is on luminal or basal side of urothelial cells. SARS-CoV-2 might cause viral cystitis through either urine from the luminal side of urothelium or from the basal side during the phase of viremia [5].

### Prostate

Angiotensin-converting enzyme 2 (ACE-2) is the receptor for cellular entry of SARS-CoV-2. ACE-2 is a TMPRSS2 in the renin–angiotensin pathway. Interestingly, TMPRSS2 is highly expressed in human prostate epithelial cells and is androgen responsive. Moreover, it is one of the known dysregulated genes in prostate cancer [6].

### Testis

In a recent case series, it has been reported that 6 out of 34 men (17.6%) reported scrotal discomfort during their course of COVID-19 infection [7].

Urologists’ knowledge about viral infection of the gonads is mainly derived from our experience with patients suffering from mumps orchitis. Orchitis is the most common complication of mumps in post-pubertal men, affecting about 20%-30% of cases within 1–2 weeks of parotitis [8].

Within the first few days of infection, the virus attacks the testicular parenchyma, leading to inflammation. The tunica albugenia is a tough barrier with subsequent rise in intra-testicular pressure leading to pressure-induced testicular atrophy. Mumps orchitis may contribute to subfertility in about 13% of patients. On the other hand, between 30 and 85% of patients with bilateral mumps orchitis experience infertility [8].

In case of COVID-19, results of the few recent studies are still controversial. In one study [7], no detectable SARS-Cov-2 by reverse transcriptase polymerase chain reaction (RT-PCR) was identified in the semen of 34 studied male patients. Thus, it appears unlikely that SARS-CoV-2 can enter into any cells in the testis (e.g., germ cells, Leydig cells, Sertoli cells, etc.) as has been hypothesized.

In another study [9], out of the 38 eligible participants who provided a semen specimen, results of semen testing found that 6 patients (15.8%) had results positive for SARS-CoV-2.

These studies are limited by the small sample size and the short subsequent follow-up. Therefore, further studies are still required.

### Men’s health

One of the most interesting features of this pandemic is about gender and how COVID-19 is affecting men and women differently. In the USA, for example, twice as many men have been dying from the virus as women. Similarly, 69% of all coronavirus deaths across Western Europe have been males. Several factors have been proposed to explain this observation [10].

#### Biological factors

- One theory is that women’s immune response to the virus is stronger. The immune response throughout life to vaccines and infections is usually more robust and effective in females as compared to males.

This is partly down to the fact that females have two X chromosomes, whereas males have only one—which is important when it comes to COVID-19. In particular, the protein by which viruses such as SARS-CoV-2 are sensed is encoded on the X chromosome. As a result, this protein is expressed at twice the dose on many immune cells in females compared to males, and the immune response to SARS-CoV-2 is therefore amplified in females [11].

- Recent research found a higher expression of *ACE2* in lung tissue in males as compared to females and a larger proportion of *ACE2* + cells in the male type II pneumocytes [12].

#### Behavioral/social factors

- Another possibility is that the difference is down to gender-based lifestyle. There are important behavioral differences between the sexes, e.g., smoking. The sex differential in smoking is especially marked in some countries such as China, where 50% of men smoke as compared to 5% in women [13] . As a result of smoking, associated comorbidities such as heart disease and chronic obstructive pulmonary disease (COPD) aggravate morbidity and mortality from COVID-19.
- Men are much less likely than women to take advantage of primary care services [14].

### Erectile dysfunction

Male sexual function can be significantly affected by stresses. Psychogenic erectile dysfunction varies from 10 to 90%. Psychogenic impotence may occur via increased sympathetic stimulation to the sacral segment of spinal cord causing inhibition of the parasympathetic nerve supply to the penis, and thereby inhibiting erection [15].

The emergence of a novel form of coronavirus causing this pandemic crisis created a rapidly evolving stressful situation [16].

This can lead to developing de novo erectile dysfunction (ED) or worsening of a previous base line condition. The first line to manage such cases is to advise the patient of ways to cope with stress [17];

- To take breaks from watching, reading, or listening to news stories, including social media. Hearing about the pandemic repeatedly can be upsetting.
- To take care of himself physically.
  - Take deep breaths, stretch, or meditate.
  - Try to eat health.
  - Regular exercise and enough sleep.
  - Avoid alcohol and drugs.
- To make time to unwind. Try to do some other activities the patient enjoys.
- To connect with others. Talk with people you trust about your concerns and how you are feeling.
- To ask for a specialist help if he is struggling to cope with his daily life.

Interestingly, phosphodiesterase 5 inhibitor (PDE5-I) represents the standard medical treatment for erectile dysfunctions. In addition, these drugs have been used in other conditions, e.g., pulmonary fibrosis or pulmonary hypertension [18].

Nitric oxide (NO) can alleviate lung injury through decreasing levels of pro-inflammatory cytokines and inhibiting leukocytes migration into the lungs. In light of the such mechanisms involved in COVID-19 infection and given previous experiences with off-label use of Sildenafil, a possible role for PDE5-I as early supplementary medication in the management of COVID19 infection may be considered [18].

### Adrenals

Autopsy studies from severe acute respiratory syndrome (SARS) original outbreak in 2003 had shown degeneration and necrosis of the adrenal glands. Virus has been identified in the adrenal glands, hinting towards its direct cytopathic effect [19].

Impact of SARS on hypothalamic pituitary axis (HPA) was first reported by Leow et al. About 40% of survivors had evidence of central hypocortisolism, majority of which resolved within a year. A prospective study evaluating serum cortisol and ACTH in patients with severe COVID-19 is currently ongoing [19].

The Waterhouse–Friderichsen syndrome (WFS) is described as acute hemorrhagic necrosis of the adrenal glands often caused by infection. Neisseriae meningitidis is the main microorganism causing WFS yet although other infectious agents are known as a possible etiologic agent [20].

Recently, few case reports have been published reporting adrenal hemorrhages as a complication of COVID-19 [20, 21] If a COVID-19 patient becomes hemodynamically unstable or developing multi-organ failure, clinicians should think of possibility of adrenal hemorrhage in their differential diagnosis [20].

## Conclusion

Impact of COVID-19 on urology practice may not be only indirect through reducing elective surgical work. Based on upcoming reports on SARS-CoV-2 organotropism, genitourinary tract can be directly involved as well. Although uncommon, we, urologist, may extrapolate our experience with viral infection in the urinary tract so as to be able to manage such possible infections and its short- and long-term consequences.

## Data Availability

Not applicable.

## Abbreviations

SARS-CoV-2: Severe acute respiratory syndrome coronavirus 2
STD: Sexually transmitted disease
ACE 2: Angiotensin-converting enzyme 2
TMPRSS2: Transmembrane serine protease 2
UTI: Urinary tract infection
RT-PCR: Reverse transcriptase polymerase chain reaction
